# The Proteolytic Activation of (H3N2) Influenza A Virus Hemagglutinin Is Facilitated by Different Type II Transmembrane Serine Proteases

**DOI:** 10.1128/JVI.02693-15

**Published:** 2016-04-14

**Authors:** Nora Kühn, Silke Bergmann, Nadine Kösterke, Ruth L. O. Lambertz, Anna Keppner, Judith M. A. van den Brand, Stefan Pöhlmann, Siegfried Weiß, Edith Hummler, Bastian Hatesuer, Klaus Schughart

**Affiliations:** a Department of Infection Genetics, Helmholtz Centre for Infection Research, and University of Veterinary Medicine Hannover, Braunschweig, Germany; b Department of Microbiology, Immunology and Biochemistry, University of Tennessee Health Science Center, Memphis, Tennessee, USA; c Department of Molecular Immunology, Helmholtz Centre for Infection Research, Braunschweig, Germany; d Department of Pharmacology and Toxicology, University of Lausanne, Lausanne, Switzerland; e Department of Viroscience, Erasmus Medical Center, Rotterdam, Netherlands; f Infection Biology Unit, German Primate Center, Göttingen, Germany

## Abstract

Cleavage of influenza virus hemagglutinin (HA) by host cell proteases is necessary for viral activation and infectivity. In humans and mice, members of the type II transmembrane protease family (TTSP), e.g., TMPRSS2, TMPRSS4, and TMPRSS11d (HAT), have been shown to cleave influenza virus HA for viral activation and infectivity *in vitro*. Recently, we reported that inactivation of a single HA-activating protease gene, *Tmprss2*, in knockout mice inhibits the spread of H1N1 influenza viruses. However, after infection of *Tmprss2* knockout mice with an H3N2 influenza virus, only a slight increase in survival was observed, and mice still lost body weight. In this study, we investigated an additional trypsin-like protease, TMPRSS4. Both TMPRSS2 and TMPRSS4 are expressed in the same cell types of the mouse lung. Deletion of *Tmprss4* alone in knockout mice does not protect them from body weight loss and death upon infection with H3N2 influenza virus. In contrast, *Tmprss2*^−/−^ *Tmprss4*^−/−^ double-knockout mice showed a remarkably reduced virus spread and lung pathology, in addition to reduced body weight loss and mortality. Thus, our results identified TMPRSS4 as a second host cell protease that, in addition to TMPRSS2, is able to activate the HA of H3N2 influenza virus *in vivo*.

**IMPORTANCE** Influenza epidemics and recurring pandemics are responsible for significant global morbidity and mortality. Due to high variability of the virus genome, resistance to available antiviral drugs is frequently observed, and new targets for treatment of influenza are needed. Host cell factors essential for processing of the virus hemagglutinin represent very suitable drug targets because the virus is dependent on these host factors for replication. We reported previously that *Tmprss2*-deficient mice are protected against H1N1 virus infections, but only marginal protection against H3N2 virus infections was observed. Here we show that deletion of two host protease genes, *Tmprss2* and *Tmprss4*, strongly reduced viral spread as well as lung pathology and resulted in increased survival after H3N2 virus infection. Thus, TMPRSS4 represents another host cell factor that is involved in cleavage activation of H3N2 influenza viruses *in vivo*.

## INTRODUCTION

Influenza viruses pose major threats to public health, as they are responsible for epidemics and pandemics resulting in high morbidity and mortality worldwide. Several pandemics, such as the Spanish flu (1918), Asian flu (1957), and Hong Kong flu (1968), caused millions of deaths in the last century (1). Currently, only two therapies, targeting the viral proteins neuraminidase and M2, are approved to treat influenza. Therefore, novel viral or host targets for antiviral strategies to block viral replication or inhibit cellular proteins necessary for the virus life cycle are urgently needed (2). In this context, host proteases are a group of very promising antiviral targets, because proteolytic cleavage of the precursor hemagglutinin (HA_0_) into HA_1_ and HA_2_ subunits by host proteases is essential for fusion of HA with the endosomal membrane and thus represents an essential step for infectivity of the virus (3, 4). Due to the potential risk of side effects after application of broadband protease inhibitors, the specific inhibition of a single enzyme would convey a huge therapeutic benefit.

The majority of influenza viruses, including low pathogenic avian and human influenza viruses, carry a single arginine (R) residue at the cleavage site. These HAs are cleaved by host trypsin-like proteases (5–8). *In vitro* studies with cultured human respiratory epithelial cells demonstrated the involvement of several membrane-associated proteases (9). Cell culture studies further identified, among others, the transmembrane serine proteases TMPRSS2, TMPRSS4, and TMPRSS11D as enzymes able to cleave the HAs of influenza virus subtypes H1 and H3 (10–12). We previously showed that deletion of *Tmprss2* in knockout mice strongly limits viral spread and lung pathology after H1N1 influenza A virus infection (13). An essential role for TMPRSS2 in cleavage activation and viral spread was also reported for H7N9 influenza A virus (14, 15). We also demonstrated that deletion of *Tmprss2* slightly reduced body weight loss and mortality in mice after H3N2 virus infection compared to those for wild-type mice but did not protect mice from lethal infections (13, 15). Therefore, it is likely that in addition to TMPRSS2, other trypsin-like proteases of the respiratory tract are able to cleave the hemagglutinin of H3 influenza viruses.

In this study, we investigated the role of *Tmprss4* in the context of influenza A virus replication and pathogenesis in experimentally infected mice. We showed that knockout of *Tmprss4* alone did not protect mice from lethal H3N2 influenza A virus infections. In contrast, *Tmprss2*^−/−^ *Tmprss4*^−/−^ double-knockout mice showed massively reduced viral spread and lung pathology and also had reduced body weight loss and mortality.

(Part of this work was performed as Ph.D. thesis work by Nora Kühn at the University of Veterinary Medicine, Hannover, Germany.)

## MATERIALS AND METHODS

### Ethics statement.

All experiments with mice were approved by an external committee according to the national guidelines of the animal welfare law in Germany (BGBl. I S. 1206, 1313 and BGBl. I S. 1934). The protocol used in these experiments has been reviewed by an ethics committee and approved by the Niedersächsisches Landesamt für Verbraucherschutz und Lebensmittelsicherheit, Oldenburg, Germany (permit number 3392 42502-04-13/1234). Mice were maintained under specific-pathogen-free conditions at the animal facilities of the Helmholtz Centre for Infection Research (HZI). Embryonated chicken eggs were purchased from Charles River Laboratories, Germany.

### Viruses, mice, and plasmids.

Original stocks of viruses were obtained from Stefan Ludwig, University of Münster (A/PuertoRico/8/34 H1N1, Münster variant [PR8M], and A/WSN/33 H1N1), from Peter Stäheli, University of Freiburg (A/PuertoRico/8/34 H1N1, Freiburg variant [PR8F], and A/Seal/Massachusetts/1/80 H7N7 [SC35M]) (16), and from Otto Haller, University of Freiburg (A/Hong Kong/01/68 H3N2) (17). The low-virulence mouse-adapted H1N1 virus PR8M and the high-virulence mouse-adapted H1N1 virus PR8F have been described previously (18). Virus stocks for all infections were prepared by infection of 10-day-old embryonated chicken eggs. *Tmprss4* mutant mice were generated by using a replacement vector targeting the *Tmprss4* gene in 129SvEv embryonic stem (ES) cells (19). Homozygous knockout mice were backcrossed to C57BL/6J mice for 10 generations to generate the B6.129-*Tmprss4*^*tm1.1Hum*^ (*Tmprss4*^−/−^) congenic mice used in our studies. Homozygous mutant mice were genotyped by PCR analysis, using a three-primer strategy (P1, 5′GGT CAG ATG TAA AAG GTA GAC3′; P2, 5′GCT AGG TTC CTT GTT CCT G3′; and P3, 5′CAT GGA TGT GAC CAT TGT GC3′) that allowed us to distinguish between wild-type (250-bp product) and knockout (500-bp product) alleles. Double-knockout B6.129S1-*Tmprss2*^*tm1Tsyk*^ *Tmprss4*^*tm1.1Hum*^ mice were generated by interbreeding of B6.129S1-*Tmprss2*^*tm1Tsyk*^ (13, 20) and B6.129-*Tmprss4*^*tm1.1Hum*^ homozygous mice. C57BL/6J mice obtained from Janvier, France, were used as controls.

### RNA isolation and purification and reverse transcription-PCR (RT-PCR) analysis.

Total RNA was prepared from lungs by use of an RNeasy Midi kit (Qiagen, Hilden, Germany) following the manufacturer's protocol. cDNA was synthesized using the Bioscript system (Bioline GmbH, Germany). Subsequently, *Taq* polymerase (REDTaq; Sigma-Aldrich) and gene-specific primers (for exon 4, primer P1 [5′CAG TTG TGT GAC GGC CAC3′]; and for exon 11, primer P2 [5′CAC AGC ATC TCA GCG GTC A3′]) were used for PCR amplification of the *Tmprss4*-specific gene fragment.

### Infection of mice and measurement of body weight and survival.

For infection experiments, female mice at the age of 8 to 11 weeks were anesthetized by intraperitoneal injection of a ketamine-xylazine solution (85% NaCl [0.9%], 10% ketamine, 5% xylazine; 200 μl per 20 g body weight). Infection was performed by intranasal application of virus solution in 20 μl of sterile phosphate-buffered saline (PBS). Subsequently, survival and body weight loss were monitored until day 14 postinfection (p.i.). In addition to mice that were found dead, mice with a weight loss of >30% of the starting body weight were euthanized and recorded as dead.

### Detection of infectious particles.

Viral loads in infected lungs were determined by use of MDCK II (Madin-Darby canine kidney II) cells for assay of the number of focus-forming units (FFU), as described earlier (21). Briefly, a 10-fold dilution series of homogenized lung tissue was incubated with preseeded MDCK II cells for 1 h at 37°C in Dulbecco's modified Eagle's medium (DMEM) containing 5 μg/ml N-acetylated trypsin (NAT) and 0.1% bovine serum albumin (BSA). After incubation, a 1% Avicell overlay prepared in DMEM containing NAT and BSA was added and incubated for an additional 24 h. Subsequently, the cells were fixed with 4% formalin, and viral particles were detected with an anti-influenza virus antibody (Virostat). For detection of nonprocessed viral particles, lung homogenates were diluted in DMEM plus 0.1% BSA without NAT. After washing of MDCK II cells, homogenates were added and incubated for 1 h at 37°C. Afterwards, the inoculants were removed and the cells were washed twice with PBS. A 1% Avicell overlay containing BSA and NAT was added, and the assay was processed according to the above protocol.

### Hematology.

For monitoring of hematological parameters, hearts were punctured and blood was collected in EDTA tubes. Numbers of lymphocytes (Lym), granulocytes (Gr), and monocytes (Mon) in the blood were determined immediately by using a VetScan HM5 hematologic system (Abaxis).

### Quantification of cytokines and chemokines in BAL fluid.

Female C57BL/6J and *Tmprss2*^−/−^ *Tmprss4*^−/−^ mice were infected with 2 × 10^3^ FFU of H3N2 influenza A virus or with PBS (for mock controls). At 3 days p.i., bronchoalveolar lavage (BAL) fluid was collected, and levels of granulocyte colony-stimulating factor (G-CSF), granulocyte-macrophage colony-stimulating factor (GM-CSF), gamma interferon (IFN-γ), interleukin-1α (IL-1α), IL-1β, IL-6, IL-10, IL-15, IL-17, IP10, KC, monocyte chemoattractant protein 1 (MCP1), macrophage inflammatory protein 1α (MIP1α), RANTES, vascular endothelial growth factor (VEGF), and tumor necrosis factor alpha (TNF-α) were measured with the MCYTOMAG-70K mouse cytokine/chemokine magnetic bead panel (Merck Millipore) following the manufacturer's instructions. Plates were read in a Luminex 100 apparatus.

### Immunohistochemical and immunofluorescence analyses and ISH.

Lungs were prepared and immersion fixed for 24 h in 4% buffered formaldehyde solution (pH 7.4), dehydrated in a graded ethanol series, and embedded in paraffin. Sections (0.5 μm) were cut from three evenly distributed levels of paraffin blocks and stained with hematoxylin and eosin. For immunohistochemical studies, sections were stained overnight at 4°C with a primary antibody against influenza A virus nucleoprotein (clone hb65; ATCC, Wessel, Germany). Subsequently, tissue sections were incubated for 30 min with horseradish peroxidase (HRP)-labeled goat anti-mouse IgG2a (Biozol) and counterstained with hematoxylin (22). Semiquantitative assessment of influenza virus antigen expression in the lungs was performed as reported earlier (23), with the following minor modifications. For the alveoli, 25 arbitrarily chosen 20× objective fields of lung parenchyma per lung slide were examined by light microscopy for the presence of influenza virus nucleoprotein, without the knowledge of the identities of the animals. The score for each animal was presented as the percentage of positive fields. For the bronchi and bronchioles, the percentage of positively staining epithelium was estimated for every slide to provide the score per animal, as follows: 0, 0% staining; 1, 1 to 25% staining; 2, 25 to 50% staining; and 3, >50% staining. For immunofluorescence analyses, 12-μm-thick cryo-sections were air dried, fixed in acetone at −20°C, and rehydrated in PBS. Slides were blocked with anti-FCR (1:500; purified rat anti-mouse CD16/CD32). Primary antibodies used were AF350-conjugated rabbit anti-TMPRSS4 polyclonal antibody (Bioss) and Cy5-conjugated rabbit anti-TMPRSS2 antibody (Antikoerper-Online). Both antibodies were checked on appropriate knockout slides to exclude potential cross-reactivity. After staining, the slides were washed with PBS, dried, and mounted with Neo-Mount (Merck, Darmstadt, Germany). Analyses were performed using a Zeiss LSM510 laser scanning microscope with a 40× oil immersion objective. For *in situ* hybridization (ISH) studies of type I alveolar epithelial cells (AECI) and type II alveolar epithelial cells (AECII), 5-μm-thick formalin-fixed, paraffin-embedded lung tissue sections were used with a QuantiGene ViewRNA ISH tissue assay kit (Affymetrix, Cleveland, OH) following the manufacturer's instructions. Type 6 (*Sftpc*; for AECII) and type 1 (*Aqp5*; for AECI) QuantiGene ViewRNA probes were generated based on Affymetrix probe sets. The GenBank accession numbers for the probe sequences are as follows: for type 6 probes (*Sftpc*), NM_011359 (region 2–784); and for type 1 probes (*Aqp5*), NM_009701 (region 239–1533). Pretreatment was performed for 10 min at 90 to 95°C, and protease digestion was performed for 20 min at 40°C. Slides were stained with Fast red for type 1 probes. Type 6 probes were detected with Fast blue. Slides were then counterstained with Gill's hematoxylin. Negative controls (antisense) were run in parallel and showed no hybridization signals (not shown) (type 6 [*Sftpc*-neg] negative control [region 23–803], covered by probe set NM_011359-N; and type 1 [*Aqp5*-neg] negative control [region 172–1122], covered by probe set NM_009701-N). Bright-field images were recorded using Aperio Scanscope and analyzed with Aperio Imagescope software. For detection of fluorescence images, a Zeiss LSM710 laser scanning microscope with a 20× objective was used.

### Histopathology.

Semiquantitative histopathological assessment of influenza virus-associated inflammation in the lung was performed as reported earlier (23), with the following modifications. For the extent of alveolitis, we scored the total number of alveoli affected in the lung slide as follows: 0, 0%; 1, 1 to 25%; 2, 25 to 50%; and 3, >50%. For the severity of alveolitis, bronchiolitis, and bronchitis, we scored the slides as follows: 0, no inflammatory cells; 1, few inflammatory cells; 2, moderate numbers of inflammatory cells; and 3, many inflammatory cells in all alveoli, bronchioles, and bronchi of the lung slide. For the presence of alveolar edema and the presence of alveolar epithelial necrosis, we reported scores as follows: 0, not present; and 1, present in the lung slide. For the overall score for pathological changes in the alveoli, the total score (scores for extent of alveolitis plus severity of alveolitis plus presence of alveolar edema plus presence of alveolar epithelial necrosis) was used. Slides were examined without knowledge of the identities of animals.

## RESULTS

### *Tmprss2* and *Tmprss4* are coexpressed in alveolar and bronchial regions.

**FIG 1:**
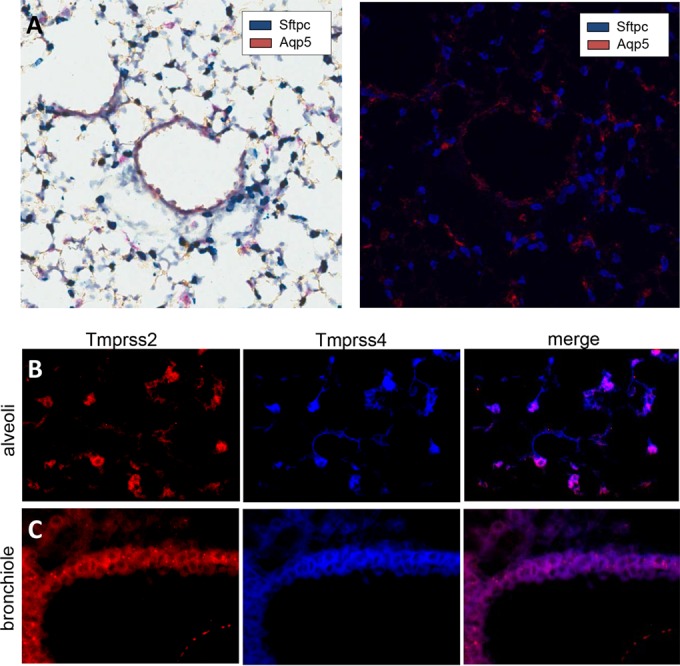
*Tmprss2* and *Tmprss4* are expressed in bronchial and alveolar regions. (A) *In situ* hybridization of lung slides from noninfected C57BL/6J lungs with probes specific for AECI (*Aqp5*) and AECII (*Sftpc*) (left, bright-field image; right, fluorescence image). Magnification, ×20. In both images, AECI (*Aqp5*) are stained red and AECII (*Sftpc*) are stained blue. (B and C) Cryo-sections of lungs from noninfected C57BL/6J mice were immunostained for TMPRSS2 (red) and TMPRSS4 (blue). Magnification, ×40. TMPRSS2 and TMPRSS4 were coexpressed in round, granular, and roughly cuboidal cells in the alveolar region (B) and in bronchial epithelial cells (C). No cross-reactivity of the antibodies was observed in appropriate knockout controls (data not shown).

It was described previously that *Tmprss2* is expressed in type 2 pneumocytes and bronchial epithelial cells (24), the main target cells for influenza A viruses (25, 26). TMPRSS4 is an additional protease with *in vitro* HA cleavage potential (10, 27). Therefore, we examined the expression profile of *Tmprss4* in mouse lung tissues. We performed *in situ* hybridization (ISH) analyses to differentiate between type I and type II alveolar epithelial cells (AECI and AECII, respectively) in noninfected mouse lungs. As shown in Fig. 1A, round and mostly cuboidal AECII were detected by expression of the cell type-specific marker surfactant-associated protein C (*Sftpc*; stained blue). Thin and flat AECI could be identified by expression of the cell type-specific marker aquaporin 5 (*Aqp5*; stained red). In addition, we used immunohistochemical staining to detect expression of both TMPRSS2 and TMPRSS4. Staining could be observed in cuboidal and granular cells of alveolar tissue, representing AECII (Fig. 1B), as well as in the bronchiolar epithelium (Fig. 1C).

### Deletion of *Tmprss4* does not affect body weight loss and viral replication in H1N1 and H3N2 virus-infected mice.

We used mice carrying a deletion in the *Tmprss4* gene to study the role of TMPRSS4 during influenza virus infection *in vivo*. RT-PCR analysis of knockout lung tissue confirmed the absence of the full-length *Tmprss4* transcript (data not shown). No protein was detected in knockout mice by immunohistochemical staining (data not shown). *Tmprss4*-deficient mice showed normal reproduction rates and development and growth patterns and had no obvious abnormal phenotype (19).

**FIG 2:**
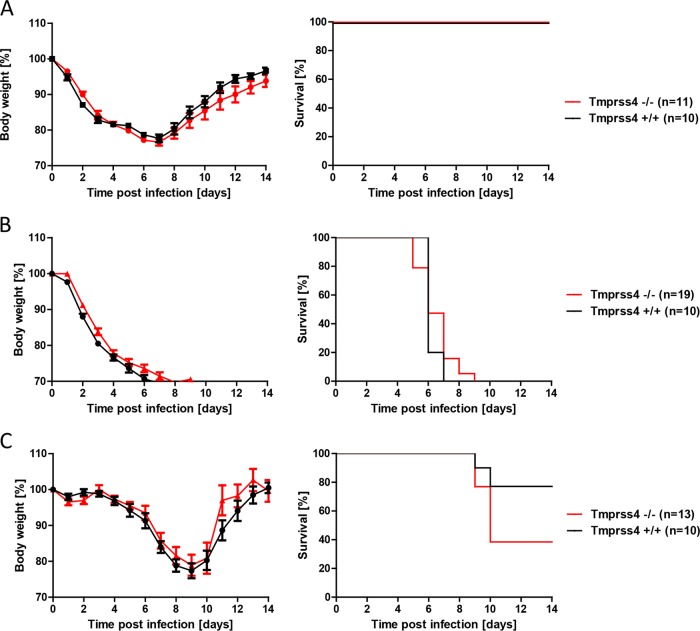
*Tmprss4*^−/−^ mice are not resistant to H1N1 and H3N2 virus infection. Eight- to 11-week-old female mice were infected with 2 × 10^5^ FFU of mouse-adapted PR8M virus (A/PuertoRico/8/34) (A), 2 × 10^3^ FFU of mouse-adapted H3N2 virus (A/HongKong/01/68) (B), or 10 FFU of H3N2 influenza virus (C) by intranasal application. Body weight loss was monitored until day 14 p.i. Mice with a weight loss of >30% of the starting body weight were euthanized and recorded as dead. Weight loss data represent mean values ± standard errors of the means (SEM). No significant differences in body weight loss and survival were observed between knockout and wild-type mice. Significances were calculated using the Mann-Whitney U test (body weight loss) and the log rank test (survival).

**FIG 3:**
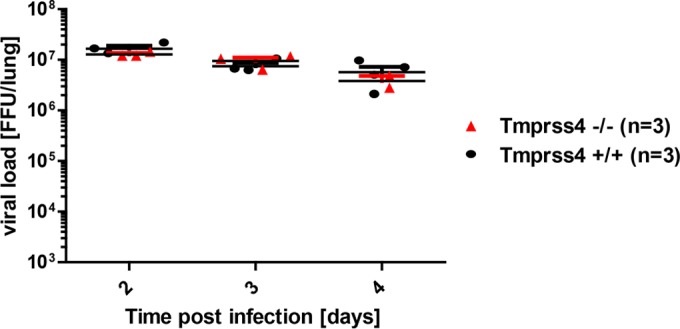
*Tmprss4* knockout mice show lung viral loads similar to those of wild-type mice after H3N2 influenza A virus infection. Eight- to 11-week-old female mice were infected with 2 × 10^3^ FFU of mouse-adapted H3N2 influenza virus by intranasal application, and numbers of infectious particles in lung homogenates were determined. Individual values, means, and SEM are presented. Viral loads were not significantly different in infected wild-type and infected homozygous mutant mice at days 2 to 4 p.i.

Wild-type as well as *Tmprss4*^−/−^ mice were infected with mouse-adapted PR8M virus (A/PuertoRico/8/34; H1N1). Both wild-type and *Tmprss4*^−/−^ mice lost weight after infection, with comparable kinetics, and showed similar survival rates (Fig. 2A). Analogous results were obtained after infection with two additional H1N1 virus variants (PR8F and A/WSN/33) (data not shown) and after infection with a multibasic, mouse-adapted H7N7 virus (A/Seal/Massachusetts/1/80) (data not shown). After infection with 2 × 10^3^ FFU of mouse-adapted H3N2 virus (A/HongKong/01/68) (17), body weight loss and survival were not different between the two mouse strains (Fig. 2B). Also, no statistically significant difference was observed after infection with a lower dose (10 FFU) of H3N2 virus (Fig. 2C). Measurement of viral loads in lungs of infected mice revealed equal viral loads in wild-type and *Tmprss4* knockout mice at days 2, 3, and 4 p.i. (Fig. 3). These results indicated that loss of *Tmprss4* in knockout mice does not protect mice from virus replication, spreading, or pathogenesis after infection with H3N2 or H1N1 influenza virus in comparison to wild-type mice.

### *Tmprss2*^−/−^ *Tmprss4*^−/−^ double-knockout mice show reduced virus replication and are protected from severe pathogenesis after H3N2 virus infection.

**FIG 4:**
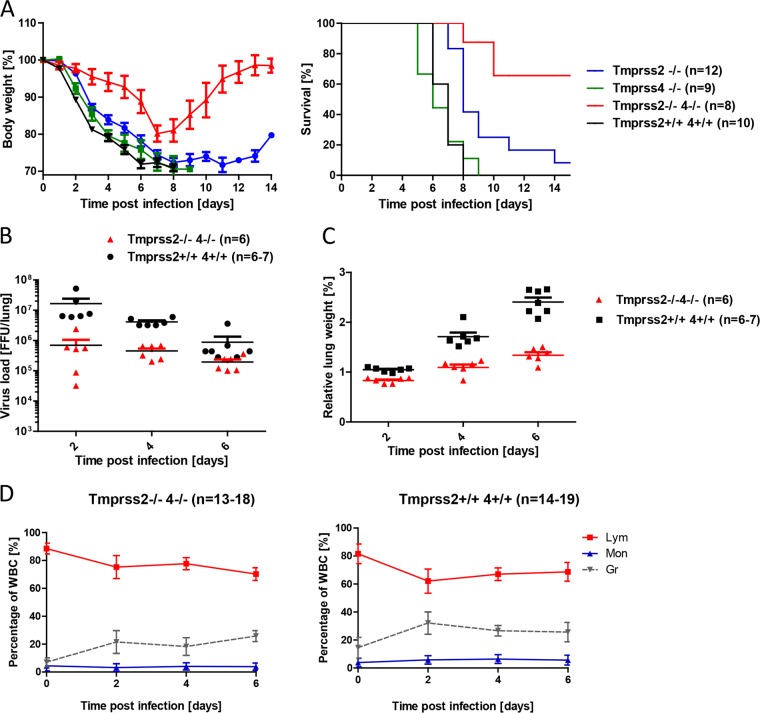
*Tmprss2*^−/−^ *Tmprss4*^−/−^ double-knockout mice show reduced body weight loss and mortality after infection with H3N2 influenza A virus. (A) Eight- to 11-week-old female mice were infected with 2 × 10^3^ FFU of mouse-adapted H3N2 influenza virus by intranasal application, and body weight and survival were monitored until day 14 p.i. In addition to mice that were found dead, mice with a weight loss of >30% of the starting body weight were euthanized and recorded as dead. (B) Numbers of infectious particles in lung homogenates were determined. Individual values, means and SEM are presented. (C) Relative lung weights were determined by weighing freshly prepared lungs and determining the percent ratio of lung weight to body weight. Individual values, means, and SEM are shown. (D) Hematological parameters were measured with a VetScan HM5 system, and the kinetics of relative numbers of lymphocytes (Lym), monocytes (Mon), and granulocytes (Gr) were determined. Homozygous *Tmprss2*^−/−^ *Tmprss4*^−/−^ knockout mice lost significantly less body weight than wild-type mice (e.g., *P* < 0.0001 at day 2 and *P* < 0.0001 at day 4; Mann-Whitney U test). *Tmprss2*^−/−^ *Tmprss4*^−/−^ mice showed significantly reduced mortality compared to that of wild-type mice (*P* < 0.0001; log rank test) as well as single-knockout mice. Viral loads were significantly higher in infected wild-type mice than in infected homozygous mutant mice at days 2, 4, and 6 p.i. (*P* < 0.01; Mann-Whitney U test). Furthermore, lung weights were significantly higher in infected wild-type mice than in infected *Tmprss2*^−/−^ *Tmprss4*^−/−^ knockout mice (*P* < 0.01). In the hemograms, lymphocyte numbers decreased until day 2 p.i., and granulocytes increased in wild-type mice on day 2 p.i. and, to a lesser degree, in *Tmprss2*^−/−^ *Tmprss4*^−/−^ knockout mice.

Both TMPRSS2 and TMPRSS4 are able to cleave H3 hemagglutinin *in vitro* and are expressed in influenza virus target cells. Therefore, we generated *Tmprss2*^−/−^ *Tmprss4*^−/−^ double-knockout mice and infected them with 2 × 10^3^ FFU H3N2 virus. Indeed, infected double-mutant mice showed a delayed and significantly reduced loss of body weight compared to wild-type mice or *Tmprss2*^−/−^ or *Tmprss4*^−/−^ single-knockout mice (Fig. 4A). Furthermore, viral loads were significantly lower in double-knockout mice than in wild-type mice after infection with 2 × 10^3^ FFU H3N2 virus at days 2 to 6 p.i. (Fig. 4B). In addition, the relative weight of wild-type lungs increased considerably more from days 2 to 6 postinfection than that of knockout lungs (Fig. 4C), indicating less infiltration of immune cells and accumulation of fluid. In agreement with these observations, the relative number of granulocytes in peripheral blood increased to higher levels in wild-type mice than in double-knockout mice after infection with H3N2 virus (Fig. 4D), which represents an indicator of severe influenza virus infection (28). However, both wild-type and knockout mice showed similar degrees of lymphopenia during the first 2 days p.i., followed by an increase of lymphocytes in both knockout and wild-type mice (Fig. 4D). Similar granulocytosis was observed in both strains on days 2 to 6 p.i.

**FIG 5:**
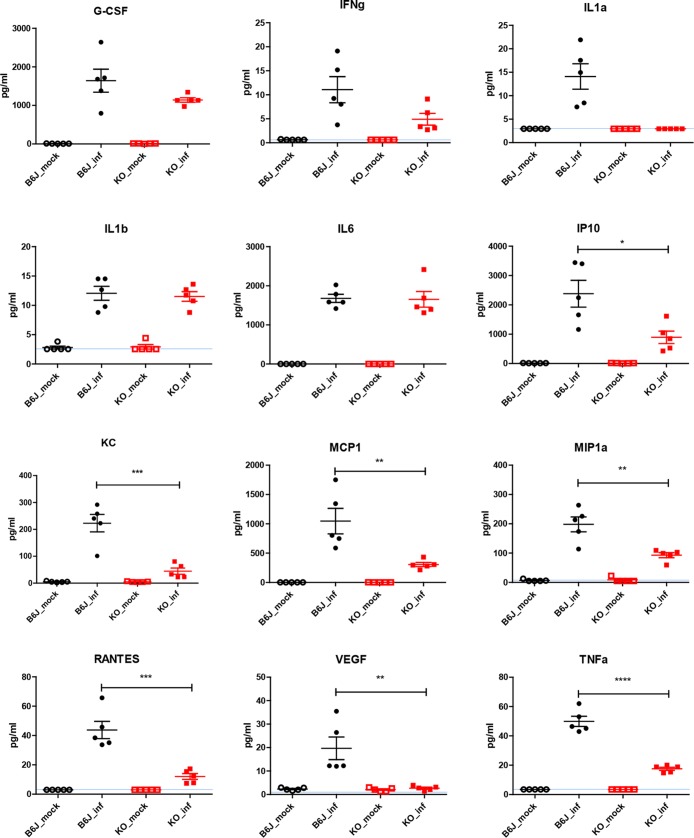
Levels of inflammatory cytokines and chemokines in BAL fluid samples from C57BL/6 and *Tmprss2*^−/−^ *Tmprss4*^−/−^ mice. Female C57BL/6J (black symbols) and *Tmprss2*^−/−^ *Tmprss4*^−/−^ (red symbols) mice were infected with 2 × 10^3^ FFU of H3N2 influenza virus, and BAL fluid was collected on day 3 p.i. PBS-treated mice were used as mock controls (open symbols). Five biological replicates were analyzed at each time point. Individual values, means, and SEM are presented. Detection limits of the individual proteins are indicated by blue lines. Samples below the detection threshold were set to the respective detection limit. Significances were calculated using the Mann-Whitney U test. *, *P* < 0.05; **, *P* < 0.01; ***, *P* < 0.001.

Furthermore, we measured the protein levels of inflammatory chemokines and cytokines in BAL fluid. Three days after infection, increases of the levels of 13 chemokines were observed in C57BL/6 mice compared to mock-infected mice. Most importantly, we observed significantly less expression of IP10, KC, MCP1, MIP1α, RANTES, VEGF, and TNF-α in knockout mice than in wild-type mice (Fig. 5). No detectable levels of IL-1α were observed in knockout samples at this time point.

By histopathology, both wild-type and knockout mice developed a multifocal mild to severe necrotizing and suppurative broncho-interstitial pneumonia (Table 1). For the wild-type mice at day 2 p.i., the alveolar septa were multifocally mildly thickened, with infiltration of few neutrophils. The alveolar, bronchiolar, and bronchial lumina contained scant to moderate amounts of inflammatory exudate, consisting of viable and degenerate neutrophils, increased numbers of alveolar macrophages, and mild accumulation of proteinaceous fluid (edema) and hemorrhage. Multifocally, there was moderate necrosis of the bronchiolar and bronchial epithelia. Around bronchioles, bronchi, and pulmonary blood vessels, there was an infiltration of moderate numbers of viable and degenerate neutrophils, lymphocytes, plasma cells, and macrophages. At 4 days p.i., the lesions were more severe and extended over a larger area of the lungs than at 2 days p.i., and they consisted of more severe infiltration by inflammatory cells, mild alveolar and moderate bronchiolar epithelial necrosis, and more intra-alveolar edema (Table 1). At 6 days p.i., the lesions were more severe and extended over a larger area than at 4 days p.i. Also, there was mild type II alveolar epithelial cell hyperplasia. For the knockout mice, the overall pathological scores in the alveoli were comparable to those for the wild-type mice at 2, 4, and 6 days p.i. However, the bronchiolar damage was less severe than that in the wild-type mice on all days.

**TABLE 1:** Scores for antigen presence by immunohistochemistry and for pathological changes for wild-type (WT) and knockout (KO) mice infected with H3N2 viruses at days 2, 4, and 6 p.i.

Group	dpi	Score			
		Alveoli		Bronchioles and bronchi	
		Antigen presence	Pathological change	Antigen presence	Pathological change
WT	2	15.3	4.3	2.7	2.3
	4	22.7	9.7	2.0	3.0
	6	23.7	14.0	1.0	2.7
KO	2	9.7	5.3	2.0	2.0
	4	15.0	9.0	1.0	1.7
	6	20.3	13.3	1.0	1.7

**FIG 6:**
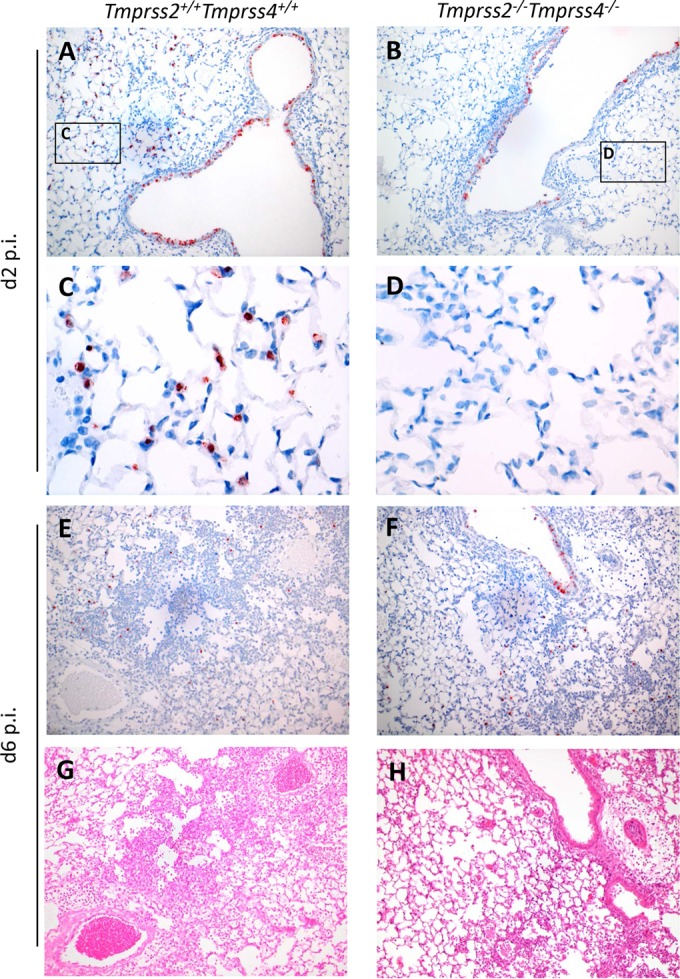
*Tmprss2*^−/−^ *Tmprss4*^−/−^ double-knockout mice exhibit milder lung pathology and reduced viral spread into alveolar regions after infection with H3N2 virus. Eight- to 11-week-old female mice were infected with 2 × 10^3^ FFU of mouse-adapted H3N2 influenza virus by intranasal application. Serial lung sections were stained on days 2, 4, and 6 p.i. with anti-influenza virus antibody and hematoxylin (A to F) or with hematoxylin and eosin (G and H). Magnification, ×10 for panels A, B, and E to H and ×40 for panels C and D. (A to D) On day 2 p.i., virus-infected cells were observed mainly in bronchiolar regions of knockout mice and in bronchiolar as well as alveolar regions of wild type mice. (E to H) Both wild-type and mutant mice showed viral spreading into alveolar regions on day 6 p.i. However, in wild-type lungs, the tissue was more densely consolidated, with larger numbers of infiltrating immune cells, than the case for *Tmprss2*^−/−^ *Tmprss4*^−/−^ knockout mice. Furthermore, hemorrhages as well as edema (G and H) were observed in infected C57BL/6J mouse lungs, indicating a more severe pathology.

By immunohistochemistry, influenza virus antigen was present in respiratory epithelial cells, with stronger expression in the nucleus than in the cytoplasm. For the wild-type mice at 2 days p.i., multifocally, few type II alveolar epithelial cells and many bronchiolar and bronchial epithelial cells were expressing virus antigen (Fig. 6A and C). At 4 days p.i., more alveolar cells were positive and fewer bronchiolar and bronchial epithelial cells were positive than at 2 days p.i. At 6 days p.i., there were more positive alveolar epithelial cells and comparable numbers of positive bronchiolar and bronchial epithelial cells compared to those at 4 days p.i. (Fig. 6E). For the knockout mice, there were fewer positive cells overall than the case for wild-type mice (Fig. 6B, D, and F). The number of positive alveolar epithelial cells increased from 2 to 6 days p.i., while the number of bronchiolar and bronchial epithelial cells decreased from 2 to 4 and 6 days p.i.

**FIG 7:**
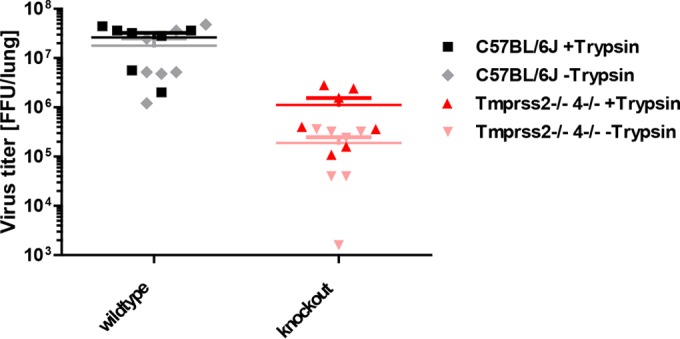
Virus isolated from *Tmprss2*^−/−^ *Tmprss4*^−/−^ lungs exhibits reduced infectivity in the absence of trypsin. Eight- to 11-week-old female mice were infected with 2 × 10^3^ FFU of mouse-adapted H3N2 influenza virus by intranasal application. On day 2 p.i., the ratio of processed to nonprocessed virus (HA cleavage or not) in lung homogenates was determined using a modified FFU assay without exogenous trypsin. The total number of virus particles (mature or not) was determined in the presence of trypsin. Samples from mutant mice showed significantly lower levels of matured viruses than the total number of virus particles (*P* < 0.05). No difference was detectable in samples from infected wild-type mice. Data shown are means ± SEM for 7 replicates from two independent experiments.

Finally, to examine whether the reduced viral replication in the lungs of mutant mice is also reflected in reduced HA activation, we performed a modified FFU assay of lung homogenates. To prevent artificial HA cleavage and following viral entry into MDCK II cells by addition of exogenous trypsin, the homogenates were incubated on the cells without additional proteinase in the medium. Under these conditions, only completely matured virus particles will enter cells and form foci. We were able to detect a significant smaller amount of matured virus than the total amount of virus in samples from double-knockout mice (Fig. 7). In contrast, no difference was observed in wild-type samples with and without trypsin treatment. Taken together, these data show that TMPRSS2 and TMPRSS4 mediate activation of H3N2 influenza A viruses by cleavage maturation of viral hemagglutinin progeny and that deletion of these host factors results in reduced viral replication, spreading, and pathogenesis.

## DISCUSSION

Seasonal influenza viruses as well as newly emerging subtypes pose a major threat to human health. Influenza viruses are dependent on host cell factors for cell entry and replication. The identification of such host factors and an understanding of their role during the influenza virus life cycle are of great importance for the development of novel therapeutic targets. In particular, host proteases that exhibit trypsin-like activity, such as TMPRSS2, TMPRSS4, TMPRSS11D (HAT), ST14 (matriptase), KLK5, KLK12, TMPRSS11E (DESC1), and TMPRSS13 (MSPL), have been shown to cleave influenza virus HAs with a monobasic cleavage site and to support multicycle virus replication in cell culture (11, 27, 29–31). Notably, altered *TMPRSS2* expression was identified as a susceptibility marker for severe A(H1N1)pdm09 and A(H7N9) influenza virus infections in humans (32). On the other hand, the trypsin-like proteases prostasin, hepsin, TMPRSS3, TMPRSS6, TMPRSS9, TMPRSS10, TMPRSS11B, and TMPRSS11F did not activate HA upon coexpression in mammalian cells (10, 31, 33).

We showed previously that TMPRSS2 is required for H1 cleavage activation *in vivo* (13). *Tmprss2*^−/−^ mutant mice are completely protected from mortality after infection with several H1N1 and H7N9 viruses (13, 14, 32). In addition, infection with 10 FFU of A/HK/01/68 (H3N2) virus, which also carries a monobasic cleavage site in HA, resulted in less body weight loss and lower mortality for *Tmprss2*^−/−^ mice than for wild-type mice. However, this difference was less pronounced after infection with an increased virus dose. Therefore, we investigated whether a further protease may be involved in cleavage activation of H3N2 viruses.

Here we showed for the first time that H3 hemagglutinin can recruit different host proteases for cleavage activation *in vivo*. Deletion of *Tmprss4* alone in single-knockout mice did not have a measurable effect with respect to body weight loss, survival, or pathology. However, deletion of both *Tmprss2* and *Tmprss4* in double-knockout mice significantly improved morbidity and survival after H3N2 virus infection. Nevertheless, the mice still showed limited body weight loss, indicating residual activity and spreading of viral particles.

Wild-type mice exhibited a higher mortality than that of the double-knockout mice and showed increased viral loads at days 2 and 4 p.i. In addition, wild-type mice exhibited higher levels of chemokines and higher lung weights at days 2, 4, and 6 p.i., which are indicative of higher rates of cellular infiltration and lung edema. Furthermore, bronchiolar damage was more severe in wild-type mice than in double-knockout mice on all days. Thus, the increased mortality rate for wild-type mice is most likely caused by a higher degree of tissue destruction by the virus at early time points. In addition, more immunopathology as a result of increased infiltration of immune cells and elevated levels of inflammatory chemokines may further contribute to increased pathology in and reduced survival of wild-type mice.

Several studies have addressed the physiological role of TMPRSS4, and three specific substrates have been identified: HA of influenza virus (10), urokinase-type plasminogen activator (PLAU) (34), and the epithelial sodium channel (SCNN1A) (35). An increased expression of TMPRSS4 was associated with increased progression and metastatic potential of several cancers (36). TMPRSS4 transcripts have been detected in the gastrointestinal tract (esophagus, stomach, small intestine, and colon), the urogenital tract (kidney and bladder), mouse bronchiolar-alveolar epithelial cells, and human lung tissue (27, 37, 38). Nevertheless, the *in vivo* function of this protein is still unclear. A recent study showed that the regulation of the epithelial sodium channel is not affected by deletion of *Tmprss4* (19). For lung tissue, TMPRSS2 expression has been described for human bronchial epithelium, AECII, and alveolar macrophages (10, 24). We confirmed the expression of *Tmprss2* and *Tmprss4* in the alveolar region and the bronchial epithelial cells of murine lungs and showed that both proteases were coexpressed in granular and roughly cuboidal cells, representing AECII. In other studies, influenza A virus antigens were detected almost exclusively in AECII and, to a lesser degree, in alveolar macrophages (25, 26). This colocalization of host proteases and virus strongly suggests that both proteases can be recruited for HA maturation.

The protease cleavage site (PEKQTR) of the mouse-adapted H3N2 variant (17) which was used in our studies is identical to the cleavage site in the original human isolate (A/Hong Kong/01/68 H3N2). In general, seasonal influenza viruses of the H3 subtype cause more severe symptoms in humans than H1 viruses. One may thus hypothesize that the increased accessibility of the H3 HA to more than two proteases allows for more efficient replication of H3N2 viruses, which may be associated with more severe pathology. The more proteases are able to cleave the hemagglutinin, the faster the virus can replicate and cause illness. It has been shown that increased expression of TMPRSS2 leads to more severe outcomes of human disease caused by the pandemic H1N1 (2009) virus or the novel avian H7N9 virus (32). Comparison of the amino acid sequences of the H1 (PSIQSR) and H3 (PEKQTR) cleavage sites shows three differences (also see Fig. 9 in reference 13). However, it is likely that specificity and efficiency for protease cleavage are determined not only by the amino acid sequence of the cleavage site but also by the overall tertiary or quaternary structure. The HA cleavage site of the H7N9 virus, which is also cleaved by TMPRSS2 (14, 15), supports this view, as it consists of the amino acids EIPKGR, which are quite different from both the H1 and H3 cleavage sites. Therefore, it will be important to investigate the structures of both proteases and hemagglutinins to determine the molecular mechanisms of HA cleavage susceptibility and efficiency.

It should be noted that deletion of *Tmprss2* did not change the tropism of H3N2 virus infection in mice (13). This is consistent with the observation that both proteases are expressed in the same cell types. Thus, after removal of *Tmprss2* alone, *Tmprss4* is still able to activate the HAs of H3N2 viruses, and *vice versa*. Survival rate was increased only after deletion of both proteases due to reduced viral loads in the lungs. Although virus replication was decreased, we still detected processed HA protein, viral spreading, and pathology. From these data, we conclude that in addition to TMPRSS2 and TMPRSS4, further proteases are able to cleave and activate the hemagglutinin of H3N2 viruses. Different proteases have been shown to activate H3 viruses *in vitro* (11, 29–31). TMPRSS11D (HAT) is expressed mainly in the murine trachea and bronchi and is thought to cleave HA mainly in the upper regions of the lower respiratory tract (14, 39, 40). Furthermore, kallikrein-related peptidase 5 (KLK5) was described to cleave H1 and H3 HAs (30), tryptase Clara (TPSB2) was reported to cleave H3 HA (5), and matriptase (ST14) cleaves H3 HA to a certain degree (29). A recent study also reported that the host proteases TMPRSS11E (DESC1) and TMPRSS13 (MSPL) are capable of cleaving H1, H2, and H3 HAs *in vitro* (31).

In summary, our *in vivo* analyses provide further important insights into the involvement of host proteases for influenza virus HA processing. Our studies suggest that an inhibitor targeting TMPRSS2 and TMPRSS4 would be well suitable to block replication of H1N1 and, to a major degree, H3N2 influenza virus infections, which represent the current seasonal subtypes in humans. It should be noted, however, that our results were obtained with a mouse model and that the development or use of any potential inhibitor will require validation studies in humans.
